# Proximity of Residence to Irrigation Determines Malaria Risk and *Anopheles* Abundance at an Irrigated Agroecosystem in Malawi

**DOI:** 10.4269/ajtmh.21-0390

**Published:** 2021-10-18

**Authors:** Charles Mangani, April N. Frake, Grivin Chipula, Wezi Mkwaila, Tasokwa Kakota, Isaac Mambo, Jerome Chim'gonda, Don Mathanga, Themba Mzilahowa, Leo Zulu, Edward Walker

**Affiliations:** ^1^ Department of Public Health, School of Public Health and Family Medicine, College of Medicine, University of Malawi, Blantyre, Malawi;; ^2^ Malaria Alert Centre, College of Medicine, University of Malawi, Blantyre, Malawi;; ^3^ Department of Geography, The University of Alabama, Tuscaloosa, Alabama;; ^4^ Center for Global Change and Earth Observation, Michigan State University, East Lansing, Michigan;; ^5^ Agricultural Engineering Department, Lilongwe University of Agriculture and Natural Resources, Lilongwe, Malawi;; ^6^ Horticulture Department, Lilongwe University of Agriculture and Natural Resources, Lilongwe, Malawi;; ^7^ Department of Basic Sciences, Lilongwe University of Agriculture and Natural Resources, Lilongwe, Malawi;; ^8^ Department of Extension, Lilongwe University of Agriculture and Natural Resources, Lilongwe, Malawi;; ^9^ Department of Agricultural Extensions Services, Malawi Ministry of Agriculture, Irrigation and Water Development, Lilongwe, Malawi;; ^10^ Department of Geography, Environment, and Spatial Sciences, Michigan State University, East Lansing, Michigan;; ^11^ Department of Microbiology and Molecular Genetics, Michigan State University, East Lansing, Michigan

## Abstract

As countries of sub-Saharan Africa expand irrigation to improve food security and foster economic growth, it is important to quantify the malaria risk associated with this process. Irrigated ecosystems can be associated with increased malaria risk, but this relationship is not fully understood. We studied this relationship at the Bwanje Valley Irrigation Scheme (800 hectares) in Malawi. Household prevalence of malaria and indoor *Anopheles* density were quantified in two cross-sectional studies in 2016 and 2017 (5,829 residents of 1,091 households). Multilevel logistic regression was used to estimate the association between distance to the irrigation scheme and malaria infection and mosquito density. The prevalence of malaria infection was 50.2% (2,765/5,511) by histidine-rich protein 2–based malaria rapid diagnostic tests and 30.1% (1,626/5,403) by microscopy. Individuals residing in households within 3 km of the scheme had significantly higher prevalence of infection (adjusted odds ratio [aOR] = 1.41; 95% confidence interval [CI] 1.18, 1.68); school-aged children had the highest prevalence among age groups (aOR = 1.34; 95% CI 1.11, 1.63). Individuals who reported bed net use, and households with higher socioeconomic status and higher level of education for household head or spouse, had lower odds of malaria infection. Female *Anopheles* mosquitoes (2,215 total; *Anopheles arabiensis*, 90.5%, *Anopheles funestus*, 9.5%) were significantly more abundant in houses located within 1.5 km of the scheme. Proximity of human dwellings to the irrigation scheme increased malaria risk, but higher household wealth index reduced risk. Therefore, multisectoral approaches that spur economic growth while mitigating increased malaria transmission are needed for people living close to irrigated sites.

## INTRODUCTION

Malaria remains a significant global health problem with an estimated 229 million cases and 409,000 deaths in 2019 alone.^1^ The risk of the disease remains high in many sub-Saharan African (SSA) countries, including Malawi. In 2019, Malawi’s estimated annual country-wide incidence of malaria was 207 per 1,000 individuals including 6,308 deaths.^1^ In the past decade, intensive use of malaria control measures has resulted in substantial reduction in disease burden: district-level prevalence for infection with *Plasmodium falciparum* declined by 47.2% from 29.4% in 2010 to 15.2% in 2017.^2^ Nevertheless, the country remains a high-burden country with meso-endemic malaria transmission risk.^2^

Variation in the spatiotemporal patterns of malaria risk and disease burden depend on, among other factors, the environment, vector ecology, and the coverage and use of control measures. Agroecological environments have generally been associated with increased malaria intensity.^3^^–^^8^ Studies have shown that the characteristics of the farming practices, crop types, proximity of households to breeding habitats, and coverage and uptake of malaria control measures affect heterogeneity in mosquito abundance and malaria risk across various agroecosytems.^9^^–^^11^ Irrigated agroecosytems produce and expand habitat characteristics favored by malaria-carrying mosquitoes and increase human exposure, resulting in an upsurge of malaria transmission, particularly among rice irrigation farming communities.^12^^–^^16^

To cope with the challenge of increasing food demand from absolute increase in population, declining soil fertility, shrinking average farm holdings, and rainfall variability, several large- and small-scale irrigation schemes have been constructed or are under construction in many countries throughout sub-Saharan Africa.^11^^,^^17^^–^^19^ In 2015, the Government of Malawi launched the Irrigation Master Plan (IMP) with the goal of expanding irrigable area from 104,298 hectares to 220,000 hectares by 2035 through the rehabilitation of older irrigation schemes and development of new ones.^20^ In addition, Malawi’s Green Belt Authority is charged with expanding irrigated agriculture under the Green Belt Initiative laid out in 2009.^21^ Despite the anticipated socioeconomic benefits of these endeavors, irrigation development risks stalling or reversing progress made in malaria reduction especially in communities residing in close proximity to irrigated agriculture.^22^^,^^23^ Previous work has attributed irrigated agricultural systems with increasing localized malaria risk.^17^^,^^22^^–^^24^ However, malaria transmission dynamics in irrigated agro-systems is still not fully understood: density of malaria vectors, including several entomological transmission parameters, have consistently been associated with proximity to irrigated agro-systems,^9^^,^^15^^,^^25^ but findings on impact of proximity to irrigated agro-systems and malaria risk are multidirectional. Increased malaria risk was associated with rice irrigation schemes in Tanzania,^13^^,^^16^^,^^26^ a cotton irrigation scheme in Sudan,^27^ irrigated vegetable production in Ghana,^28^ and irrigation schemes in Kenya^29^ and Ethiopia.^30^^,^^31^ However, some studies have shown lower malaria prevalence in villages located near irrigated areas compared with villages near nonirrigated areas.^9^^,^^32^ This observed heterogeneity and its underlying causes need to be studied further, if the context-specific effects of irrigation are to be generalized. *Plasmodium* infection has also been observed to vary spatially even between sub-villages that are closely located.^26^^,^^33^ However, there is no study that has quantified the risk of malaria in relation to distance of human residence to irrigated agricultural areas, nor on how the socioeconomic gains provided by enhanced crop production might balance malaria risk. Furthermore, studies that have collected data on both entomological parameters and malaria prevalence are few. This study was carried out to determine the relationship between proximity of human dwellings from agricultural plots in a rice irrigation scheme and household mosquito population, and ultimately malaria risk among a rural Malawian community. We also aimed to evaluate the effect of other factors that may mediate malaria transmission dynamics in this ecosystem such as long-lasting insecticide-treated net (LLIN) access and use, level of education, socioeconomic status, and quality of housing.

## MATERIALS AND METHODS

### Study area.

**Figure 1.**
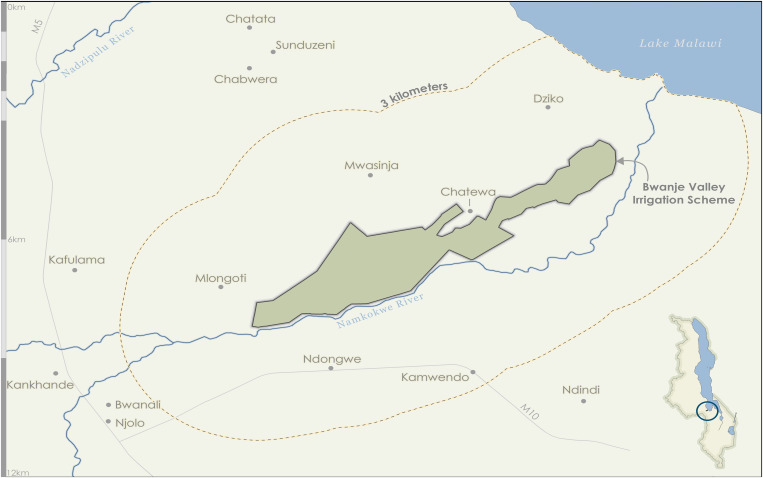
Map of the Bwanje Valley Irrigation Scheme and participating villages. Inset, Malawi showing location of the scheme in the circle. This figure appears in color at www.ajtmh.org.

This study was carried out in the Dedza district in east central Malawi at the Bwanje Valley Irrigation Scheme (BVIS) (Figure 1). BVIS is in the Lake Malawi lakeshore plain, a high malaria transmission setting. Mean annual rainfall is approximately 867 mm and unimodal, falling mainly between November and May—925 mm and 750 mm were reported in the 2016 and 2017 rainy seasons, respectively (M. Tarsizio, pers. commun., 2020). The mean maximum temperature is 27.3°C (September–December) and the mean minimum temperature 17.2°C (May–July).^34^ The scheme was established in 2000 through a cooperative agreement between the Malawi and Japanese governments. Costing US$15 million and targeting 12,000 smallholder farming households, the scheme’s goal is to improve household food security and incomes.^35^^,^^36^ The irrigated area covers some 800 hectares of land for the primary cultivation of rice during the rainy season, and horticultural crops including maize, soybean, and cowpea during the dry season. The scheme is irrigated by use of open gravity-fed irrigation canals from water diverted from Namikokwe (locally, Namkokwe) River. During the 2016 growing season, 2,067 farmers participated at BVIS from 14 surrounding villages.^37^ Primary malaria control measures in Malawi are case management and vector control through distribution and use of LLINs. A national mass distribution was completed in the study area in November 2014, with one LLIN distributed for every two household members. Indoor residual spraying has not been conducted in this area.

### Study design.

Two cross-sectional surveys of 14 villages located within a 6-km radius of BVIS were conducted at the end of the rainy season in April 2016 and April 2017. A study in Burkina Faso found that the average flight range of *Anopheles gambiae* s.s., one of the main mosquito vector species in Malawi,^38^ was less than 1.0 km^39^ and maximum flight distance is 1.7 km.^40^ Thus, a 6-km sampling distance exceeds three times the estimated maximum flight distance. Based on the decay of the *Anopheles* vector flight distance, it was estimated that between 40% and 50% of sampled houses were outside of the flight range of *Anopheles* vectors produced by the irrigation scheme. The spatial arrangement of the study villages relative to distance from the scheme provides a control population to test for impact of the irrigation scheme. Eligible participants were defined as all individuals aged 6 months or older who slept in the house for at least 2 weeks of the previous month. To estimate the minimum number of individuals required for the survey, the malaria parasite prevalence was assumed to be 25% in households furthest from the irrigation scheme.^41^^,^^42^ The sample size required for 80% power to detect a 7% difference in malaria parasite prevalence among individuals in households located < 3 km from those individuals residing in households ≥ 3 km from the scheme at a two-sided alpha of 5% was 1,291 individuals. Adjusting for a design effect of 2 and a 10% nonresponse rate, enrollment was planned for 2,869 individuals. The average household size in Malawi was 4.3 persons according to the 2008 national census data^43^; therefore, a total of 663 households were planned to be enrolled. Sampling of recruited households took place in two stages: first, villages were selected in two strata at distance buffers of 1) < 3 km and 2) 3–6 km from BVIS. Using simple random sampling, six villages were selected from the < 3 km stratum, and eight villages from the 3–6 km stratum. Villages in the 3–6 km area were oversampled because of observed lower population densities compared with those within 3 km of BVIS. The number of households sampled from each village was estimated using probability proportionate to size (i.e., total number of households within a village). Households were selected randomly for sampling from a household listing provided by the village leadership.

### Data collection.

#### Demographic and socioeconomic information of the study participants.

Questionnaires were adapted from the standardized Demographic and Health Surveys (DHS) Malaria Indicator Survey (MIS) tools and administered to the household head or a consenting adult caretaker. Survey questionnaires were designed to capture information on demographic and socioeconomic characteristics, including age, sex, history of recent (within the last 48 hours) or current fever, malaria-related care-seeking behavior, use of antimalarial medication in the past 2 weeks and source of treatment, education level, household characteristics, and household LLINs ownership and individual use. Household characteristics included roof, wall, and floor materials and were coded as natural, rudimentary, and finished, according to the MIS classification. The location of each household was collected using a handheld Global Positioning System (GPS) device. Household distances to BVIS were calculated in ArcMap^™^ 10.7.1, according to a digitized perimeter of the scheme from Frake et al. (2020).^37^

#### Malaria diagnosis.

A finger prick blood sample was obtained from all eligible participants aged 6 months or older. Malaria testing was performed using a malaria rapid diagnostic test (mRDT) (histidine-rich protein 2 [HRP-2] *P. falciparum* rapid diagnostic test SD Bioline malaria Ag Pf HRP-2; Gewerbestrasse, Switzerland). Thin and thick blood smears were prepared in the field and transported to the Malaria Alert Center laboratory in Blantyre, Malawi. Smears were stained with Giemsa and examined with a binocular microscope with an oil immersion lens to quantify the parasitemia according to published quality control methods.^44^ Two laboratory technicians read the blood slides to determine parasitemia. In cases of nonagreement, a third technician read the slides. A blood smear was considered negative if no malaria parasite was seen after scanning at least 200 fields. In compliance with College of Medicine Research and Ethics Committee guidelines, we provided malaria treatment of all individuals with positive mRDT results. A fixed combination of artemisinin-based antimalarial treatment was given to all participants with a positive mRDT result as per national guidelines. Axillary temperature was measured for all available household members.

#### Mosquito collection and identification.

Indoor adult mosquitoes were sampled from participating households within 48 hours of the malariometric survey using CDC light traps (John W. Hock, FL). Light traps were hung beside one sleeping space in each household between 18:00 hour to 06:00 hour. After collection, mosquitoes were sorted and counted by genus: *Culex* and *Anopheles*. *Anopheles* mosquitoes were subsequently sorted by sex and abdominal condition (unfed, fed), and species identification was done morphologically.^45^^,^^46^ Specimens identified as *Anopheles gambiae* s.l. were identified to species within the complex using the procedure from Walker et al. (2007).^47^ DNA was extracted from a single mosquito leg, sonicated for five minutes in buffer in wells of 96-well polymerase chain reaction (PCR) plates. Identification of species was done by automated, quantitative PCR method based upon TaqMan^™^ single nucleotide polymorphism genotyping.

#### Data analysis and statistical methods.

Malaria parasitemia, our primary outcome, was determined on the diagnosis of infection with *P. falciparum* by microscopy or malaria rapid test. Secondary outcomes were anemia and female *Anopheles* counts collected per night, per trap. The main predictor variable of interest was distance of the household from the irrigation scheme boundary. Malaria infection prevalence levels were compared between households < 3 km from BVIS and households ≥ 3 km from the scheme in addition to demographic and socioeconomic factors listed below. Symptomatic malaria infection was defined as malaria infection (microscopy) with measured fever (temperature ≥ 37.5°C) or report of fever within the past 48 hours.

Several variables were considered as potential confounders in the analysis. Individual variables included sex, age, and fever in the last week, access and use of LLINs. Household variables included house construction materials, highest level of education of household head, number of individuals in the house, and socioeconomic status based on a wealth index. Age categories were young children, 6 months to 5 years old; school-age children (SAC), 6–15 years old, and adults aged 15 years and older. LLIN use was defined as an individual living in a household that owned at least one LLIN and who slept under an LLIN the previous night. Household wall construction was coded as natural, rudimentary, or finished according to the MIS tool; roof construction was dichotomized as either natural or finished. Household wealth was differentiated by principal components analysis of household asset ownership, household infrastructure, and basic amenities like sanitation.^48^ Variables with very low frequencies (< 1%), were excluded because of their low capacity for differentiating households from each other. The wealth index was divided into quintiles (poorest, poorer, medium, wealthier, and wealthiest).

Frequencies and percentages were used to summarize data and to explore the differences in malaria positivity by distance from BVIS, demographic, and socioeconomic variables; χ^2^ tests were used for comparison. Cuzick’s test of trend^49^ was used to determine ordered effect of household distance from the scheme on malaria infection prevalence. The effects of distance from irrigation scheme, individual, and household level characteristics on malaria risk were analyzed using multilevel logistic regression models based on Generalized Estimating Equations (GEE) to account for the data hierarchy and correlated observation within households. Results from the GEE were reported as the adjusted odds ratio (aOR) and 95% confidence interval (CI). Adult mosquito density was expressed as the mean number of adult mosquitoes per light trap per night and were compared between the households at <1.5 km radius, 1.5–3 km radius, and households located ≥ 3 km from BVIS using Wilcoxon Signed Ranks Test. Variables with *P* values < 0.10 in univariate models were eligible for inclusion in the final multivariate model. The level of statistical significance was set at the stricter *P* values below 0.05 in the multivariate model. All statistical analyses were carried out using STATA (version 15.1, StataCorp, College Station, TX).

### Ethical considerations.

The study was approved by the University of Malawi’s College of Medicine Research and Ethics Committee and Michigan State University’s Institutional Review Board. Permission to conduct this study was given by the Dedza District Assembly and the BVIS governance committee. Participants were given detailed information on the study contents and procedures by the study team and local community health workers in the local language. A consent form in the local language was also provided and was explained to the participants in detail. A signed consent form was required for inclusion of a subject in the study. Informed consent was obtained from adult individuals. For individuals who were < 18 years, an assent in addition to the informed consent from their parents/guardians was obtained.

## RESULTS

### Household and study participants.

There were 1,326 households identified for participation in both surveys, of which 1,091 (82.2%) consented to participate; 537 from survey one and 554 from survey two. There were 163 households that participated in both surveys. Of the 5,829 household members enrolled to participate in the two surveys, 5,511 (95%) and 5,403 (93%) were successfully tested for malaria infection using mRDT and microscopy, respectively. A total of 741 (13.4%) individuals were tested for malaria from both surveys. Approximately 54% of survey participants were female; 15.2% were children aged less than 5 years, 34.7% were school-aged children (SAC; ages 5–15 years), and most (50%) were adults aged over 15 years (Table 1). A total of 3,505 (64.5%) individuals from 669 households were successfully tested for malaria and lived < 3 km from BVIS.

**Table 1:** Characteristics of the human study population by malaria infection status at Bwanje Valley Irrigation Scheme, Malawi, 2016–2017

Characteristic	Microscopy positive, *N* (%)	Microscopy negative, *N* (%)	Univariate unadjusted *P* value
**Individual-level factors**			
Distance residing from irrigation scheme			
Individuals living < 3 km	1,121 (32.0)	2,384 (68.0)	< 0.0001
Individuals living ≥ 3 km	505 (26.6)	1,393 (73.4)	–
Gender			
Female	864 (29.8)	2,032 (70.2)	0.654
Male	762 (30.4)	1,745 (69.6)	–
Age			
Children younger than 5 years	235 (28.5)	589 (71.5)	< 0.0001
School-aged children (age 5–15 years)	653 (34.8)	1,224 (65.2)	–
Adult (age > 15 years)	738 (27.3)	1,963 (72.7)	–
LLIN access/use			
Own at least one LLIN			
No	442 (30.5)	1,006 (69.5)	0.657
Yes	1,181 (29.9)	2,769 (70.1)	–
Own at least one LLIN for every two people			
No	1,430 (30.6)	3,240 (69.4)	–
Yes	196 (26.7)	537 (73.3)	0.033
Slept under an LLIN last night			
No	595 (34.6)	1,124 (65.4)	< 0.0001
Yes	974 (28.1)	2,496 (71.9)	–
**Household-level factors**			
Household wealth index			
Poorest	409 (35.2)	752 (64.8)	< 0.0001
Poorer	428 (30.9)	959 (69.1)	–
Medium	145 (28.1)	371 (71.9)	–
Wealthier	286 (29.8)	673 (70.2)	–
Wealthiest	235 (23.9)	749 (76.1)	–
Highest level of education of household head or spouse, *n* (%)			
None	175 (33.0)	356 (67.0)	< 0.0001
Primary school	1,188 (31.5)	2,580 (68.5)	–
Secondary school or higher	183 (22.4)	633 (77.6)	–
House wall material			
Natural	15 (38.5)	24 (61.5)	< 0.0001
Rudimentary	657 (33.3)	1,315 (66.7)	–
Finished	874 (28.1)	2,232 (71.9)	–
House roof material			
Natural	1,268 (31.0)	2,824 (69.0)	0.068
Finished	273 (28.0)	702 (71.0)	
**Survey**			
Rainy season 2016	900 (32.2)	1,894 (67.8)	< 0.0001
Rainy season 2017	726 (27.8)	1,883 (72.2)	–

LLIN = long-lasting insecticide-treated net.

### Prevalence of malaria.

**Figure 2.**
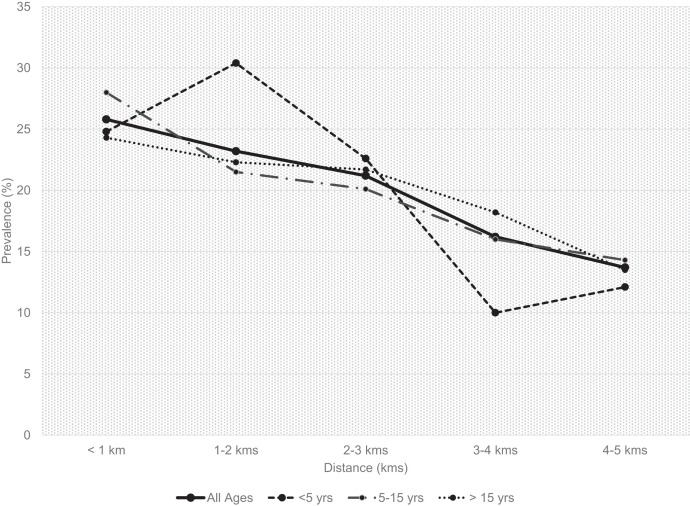
Household prevalence of *Plasmodium falciparum* infection, measured by microscopy, by distance of house from the Bwanje Valley Irrigation scheme, Malawi.

The overall prevalence of malaria infection was 50.2% by mRDT and 30.1% by microscopy. Several individual level factors were associated with microscopy positivity in univariate analyses. Malaria infection rate was significantly higher among individuals residing in households closer to the irrigation scheme (< 3 km) than those further from it (32.0% versus 26.6%, *P* < 0.0001, Table 1). Trend by household distance from BVIS was positive and highly significant, with individuals residing in households nearer the irrigation scheme having the highest malaria risk (Cochrane-Armitage Test for trends of proportion *P* value = 0.001, Figure 2). SAC aged 5–15 years were more likely to have malaria parasitemia (34.8%) than children younger than 5 years (28.5%), and adults > 15 years old (27.3%; *P* < 0.001) (Table 1). In general, 8.5% (*N* = 138/1,626) of the study participants infected with malaria parasites had gametocytes and 9.9% (152/1,533) had fever at the time of the visit.

Proportionally, significantly fewer malaria positive individuals were observed in households with the highest wealth status (23.9%) compared with individuals in the poorest households (35.2%; *P* < 0.0001). Furthermore, malaria infection prevalence was highest among individuals from households with lower levels of education. For individuals from households whose head or the spouse had at least a secondary level education, 22.4% had malaria infection compared with 33% for individuals from households whose head/spouse had no formal education (*P* < 0.0001). Malaria positive individuals also tended to live in poorer quality housing; 28% of individuals sleeping in homes with finished wall construction had malaria infection compared with 31% sleeping in homes with natural wall construction (*P* < 0.0001).

### Reported ownership and use of mosquito nets.

A total of 69.7% (760/1,091) of households reported ownership of at least one LLIN and 18.0% (196/1,091) owned one net for every two household members. There were no differences in malaria parasitemia between individuals residing in households with at least one LLIN compared with those without an LLIN (30.5% versus 29.9%, *P* = 0.605, Table 1). Conversely, individuals residing in households that owned at least one net for every two individuals were significantly less likely to have malaria parasitemia compared with those that did not (26.7% versus 30.6, *P* = 0.033, Table 1). Just over two thirds (66.6%) of individuals reported use of mosquito net during the previous night. Study participants who reported not using an LLIN the previous night had a higher prevalence of malaria infection compared with those who reported using LLINs (34.6% versus 28.1%, *P* < 0.001, Table 1).

### Determinants of malaria infection.

In a multivariable GEE analysis, several individual and household characteristics were associated with malaria infection (Table 2). Proximity of individual’s residence from BVIS, age, use of LLINs, socioeconomic status, and education of household head or spouse were significant determinants of malaria infection. Individuals residing in households < 3 km of BVIS had 41% higher odds of malaria infection than those residing ≥ 3 km from the scheme (aOR = 1.41; 95% CI 1.18,1.68). SAC showed a higher vulnerability to malaria infection with a 34% higher risk of malaria infection compared with children younger than 5 years (aOR = 1.34; 95% CI 1.11, 1.63). Factors protective against malaria infection included sleeping under a net the previous night, higher socioeconomic status, and at least secondary school level of education for the household head or spouse. Sleeping under an LLIN reduced malaria risk by 31% (aOR = 0.69; 95% CI 0.59, 0.83). Compared with individuals from the poorest households, individuals from households with the highest wealth index had 36% lower odds of malaria infection (aOR = 0.64; 95% CI 0.50, 0.81). The odds of malaria infection were also lower among individuals from households whose head or spouse had secondary or higher education compared with no formal education (aOR = 0.62, 95% CI 0.46,0.85). Lastly, survey year was significantly associated with the rate of infection; risk of malaria was 26% lower during the 2017 survey than the survey in 2016 (aOR = 0.74; 95% CI 0.64, 0.87).

**Table 2:** Risk factors for malaria infection in the human population at Bwanje Valley Irrigation Scheme, Malawi, 2016–2017

	Bivariate				Multivariable		
	Unadjusted OR	[95% CI]	*P* value	Adjusted OR		[95% CI]	*P* value
**Individual-level factors**							
Distance residing from irrigation scheme							
Individuals living ≥ 3 km	–	–	–	–		–	–
Individuals living < 3 km	1.30	[1.10, 1.52]	0.002	1.41		[1.18, 1.68]	< 0.0001
Gender							
Female	–	–	–	–		–	–
Male	1.03	[0.91, 1.16]	0.660	–		–	–
Age							
Children younger than 5 years	–	–	–	–		–	–
School-aged children (age 5–15 years)	1.35	[1.12, 1.60]	0.001	1.34		[1.11, 1.63]	0.002
Adult (age > 15 years)	0.94	[0.80, 1.11]	0.483	0.93		[0.78, 1.12]	0.479
Net access/use							
Own at least one LLIN	–	–	–	–		–	–
No	–	–	–	–		–	–
Yes	0.97	[0.82, 1.14]	0.681	–		–	–
Own at least one LLIN for every two people							
No	–	–	–	–		–	–
Yes	0.83	[0.66, 1.03]	0.090	–		–	–
Own at least one net for every two people	0.83	[0.66, 1.03]	0.093	–		–	–
Slept under a net last night							
No	–	–	–	–		–	–
Yes	0.70	[0.63, 0.86]	< 0.0001	0.69		[0.59, 0.83]	< 0.0001
**Household-level factors**							
Household wealth index							
Poorest	–	–	–	–		–	–
Poorer	0.82	[0.66, 1.02]	0.070	0.80		[0.65, 1.00]	0.046
Medium	0.72	[0.54, 0.95]	0.021	0.74		[0.56, 0.99]	0.041
Wealthier	0.78	[0.62, 0.99]	0.041	0.80		[0.62, 1.01]	0.070
Wealthiest	0.58	[0.46, 0.73]	< 0.0001	0.64		[0.50, 0.81]	< 0.0001
Highest level of education of household head/spouse							
None	–	–	–	–		–	–
Primary school	0.94	[0.75, 1.18]	0.572	0.93		[0.74, 1.17]	0.532
Secondary school or higher	0.59	[0.44, 0.79]	< 0.0001	0.62		[0.46, 0.85]	0.002
Household wall material							
Natural	–	–	–	–		–	–
Rudimentary	0.80	[0.39, 1.63]	0.539	–		–	–
Finished	0.63	[0.31, 1.28]	0.199	–		–	–
Household roof material							
Natural	–	–	–	–		–	–
Finished	0.87	[0.71, 1.05]	0.147	–		–	–
**Survey**							
Rainy season 2016	–	–	–	–		–	–
Rainy season 2017	0.81	[0.70, 0.94]	0.005	0.74		[0.64, 0.87]	< 0.0001

CI = confidence interval; LLIN = long-lasting insecticide-treated net; OR = odds ratio.

**Table 3:** Abundance (mean =/− SEM) of *Anopheles* mosquitoes caught indoors in CDC light traps, by distance from Bwanje Valley Irrigation Scheme, Malawi

	Distance of house from the scheme			
Species	≤ 1.5 km	1.5–3 km	≥ 3 km	Total
*Anopheles arabiensis*	6.8 (4.1–9.5)	5.1 (2.5–7.7)	0.8 (0.2–1.3)	4.0 (2.7–5.2)
*Anopheles funestus*	0.6 (0.3–0.8)	0.5 (0.2–0.8)	0.2 (0.0–0.4)	0.4 (0.3–0.6)
*Culex* spp.	1.5 (0.8–2.2)	1.4 (0.8–2.0)	1.2 (0.4–2.0)	1.3 (0.9–1.8)
All *Anopheles* mosquitoes	7.4 (4.6–10.2)	5.6 (2.9–8.3)	1.0 (0.3–1.7)	4.4 (3.1–5.6)

SEM = standard error of the mean.

### Abundance of malaria vectors and species composition.

**Figure 3.**
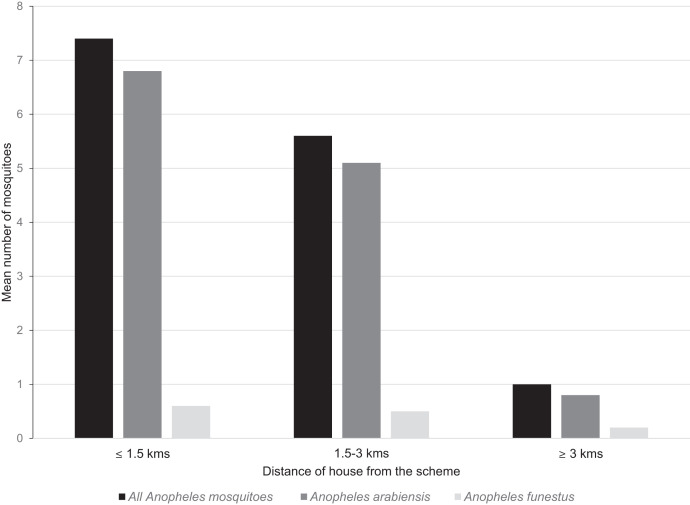
Mean abundance of *Anopheles* mosquitoes in households by distance from the Bwanje Valley Irrigation scheme, Malawi.

There were 2,888 female mosquitoes collected in 505 houses; 2,005 (69.4%) were *Anopheles gambiae* s.l., 673 (23.3%) were *Culex* sp., and 210 (7.3%) were *Anopheles funestus* (Figure 3). Of 14 *An. gambiae* s.l. complex females, all were *Anopheles arabiensis* when identified by PCR. The combined density of malaria vectors (mean number of anophelines per light trap per night) was 4.4 mosquitoes per house per night (95% CI 3.1–5.6) (Table 3). Mosquito density decreased with distance from the scheme. The density of anophelines was significantly higher (*P* < 0.001) in the residences < 1.5 km of BVIS (mean = 7.4 anophelines/trap/night; 95% CI = 4.6–10.2) than those in the 1.5–3 km (mean = 5.6 anophelines/trap/night; 95% CI = 2.9–8.3) and above the 3 km radius (mean = 1.0 anophelines/trap/night; 95% CI = 0.3–1.7, Figure 3) of the scheme.

## DISCUSSION

Our study findings demonstrate that irrigated rice agro-systems are associated with increased malaria risk in a context where malaria prevalence is already high. Furthermore, this relationship varies spatially. The distance of human dwellings to BVIS significantly influences malaria risk among household members. BVIS is an example of a prototypical smallholder irrigation scheme in sub-Saharan Africa. It is reasonable to assume that the findings of this study may be extrapolated to existing flooded, irrigated rice schemes throughout the region, and that new schemes planned for development will similarly elevate localized malaria prevalence. Although some studies have shown higher malaria prevalence for those living in households in close proximity to traditional flooded rice schemes,^14^^,^^16^^,^^31^ our findings demonstrate that the BVIS scheme added to the burden of malaria with an obvious distance decay: malaria infection prevalence declined with distance of human dwellings to the scheme. Irrigated schemes provide a collection of probable larval sites within a clearly defined geographical area. Flooded rice farming creates favorable habitats for mosquito breeding, in turn increasing exposure of individuals to infectious bites.^10^^,^^12^^,^^15^ Unsurprisingly, households closest to larval sites have been shown to have higher exposure to mosquito bites and greater risk of malaria^25^^,^^50^^,^^51^ as the dispersion distance of adult *Anopheles* mosquitoes is limited.^25^^,^^52^^–^^54^ Irrigation schemes are not homogenous in their production of larval sites; crop type, irrigation timing and intensity, maintenance, and drainage collectively define microecological zones that favor or restrict mosquito breeding and in turn influence the distribution of malaria risk for surrounding communities.^37^

The prevalence of malaria varied strongly between age groups with SAC, age 5–15 years having the highest prevalence of malaria infection compared with other age groups. Previous studies from Malawi^55^^,^^56^ and other African countries^57^^–^^59^ have demonstrated that this age group has the highest *P. falciparum* infection prevalence. Among SAC, infections tend to be asymptomatic and are less likely to be treated compared with those in younger children^55^; access and use of LLINs is also lower in this age group compared with the others.^55^^,^^56^ This partly explains the higher infection prevalence among this age group, but also indicates that SAC could contribute substantially to endemic transmission.^55^

Households with the highest wealth status and higher education of household head or spouse had significantly lower risk of malaria. The relationship between malaria risk and wealth status has been described previously and is mediated by a number of factors including access to malaria control measures (LLINs), access to treatment of illnesses, and better housing quality that limits human vector contact.^60^^–^^62^ It is prudent to note that the measure of wealth here is relative to the particular context and not a measure of absolute wealth, thus householders who are relatively better off socioeconomically have reduced risk of malaria in general, even if the wealthiest group is still poor. Lower malaria risk in households where the household head or spouse had secondary school education or higher might be linked to wealth status and the associated better standard of living,^63^ and/or better health knowledge resulting in higher levels of access and use of malaria control measures. These findings are consistent with other studies from sub-Saharan Africa.^59^^,^^64^ Our findings suggest that the economic benefits of participating as a smallholder farmer at BVIS could yield malaria protection outcomes. This along with the protective effects of education are potential targets for indirect but evidence-based malaria interventions.

Our findings did not show a significant reduction in malaria risk associated with better housing quality. Therefore, it is unlikely that the protective effect of relatively more household wealth is the result of better housing alone. Generally, the housing quality for dwellings surrounding BVIS is poor. However, a recent meta-analysis from 21 studies from sub-Saharan Africa countries conducted between 2008 and 2015 found that modern housing was associated with a 9–14% reduction in the odds of malaria infection compared with traditional housing.^65^ There has been an incremental improvement in quality of housing across sub-Saharan Africa as socioeconomic standards have improved. However, human behaviors including timing of sleep and wake patterns, and anthropophilic tendencies of the primary *Anopheles* vectors could also affect the relationship between quality of housing, mosquito densities, and malaria transmission risk. Additional work is needed to understand the complex relationship between house improvement, human and vector behaviors, and the spatial risk distribution of malaria within an irrigated agroecosystem.

From our entomological analyses, we observed spatial heterogeneity in the abundance of indoor *Anopheles* mosquitoes following a similar pattern of malaria infection prevalence. However, household *Anopheles* abundance declined at a greater rate with distance from the scheme than did household malaria infection prevalence. This was also observed in Gambia by Clarke et al. (2002).^51^ The most abundant species in our study was most likely *An. arabiensis*, a common inhabitant of rice fields in sub-Saharan Africa and a member of the *An. gambiae* s.l. complex and known to be present in the lake plain region along Lake Malawi.^66^ These findings suggest that the observed increased malaria risk from households near the scheme was mediated by the elevated abundance of *Anopheles* mosquitoes. Flooded rice farming practice is known to provide suitable breeding sites for *Anopheles gambiae* in sub-Saharan Africa.^9^ Furthermore, mosquito abundance has been observed to markedly vary in space even over a few kilometers.^12^^,^^15^ Variation in mosquito abundance over space has been associated with heterogeneity in biting rates and annual entomological inoculation rates and is higher in communities in irrigation ecosystems.^4^^,^^15^^,^^31^

There was a positive association between year of study and malaria risk. In 2017, there was a dry spell in the latter half of the rainy season in Malawi. At that time BVIS had no reservoir. The reduced volume from the Namikokwe River limited surface water within the scheme, affecting the formation and persistence of potential mosquito breeding sites. Mosquito breeding and development require an optimum combination of temperature and humidity and influences the pattern and levels of malaria infection.^67^^–^^69^

The present study was not conducted without limitations. Entomological data are only available for the 2016 season. As our findings show, survey year significantly affected malaria infection prevalence. In addition, sensitive molecular methods such as PCRs in malaria infection determination were not used in this study. Consequently, it is reasonable to assume that light malaria infection that could not be picked by microscopy and rapid diagnostic tests may have been missed.^70^ Despite these limitations, the relationship between proximity to the irrigation scheme and malaria risk was clearly established, and the socioeconomic and age-specific factors affecting risk revealed.

## CONCLUSION

The findings of this study demonstrate that distance of human dwellings to irrigated agriculture influences indoor *Anopheles* mosquito abundance and risk of malaria infection despite intensive malaria control and bed net usage. Further, this study reveals important effects of socioeconomics on malaria epidemiology; there is a linear relationship between the household wealth index and malaria risk. As the number and sizes of irrigation schemes expand in Malawi and the sub-Saharan region to boost economic growth and mitigate food insecurity concerns, it becomes important to consider how these types of economic improvements could affect malaria risk, particularly for those living in close proximity to irrigated sites. Appropriate control measures to curb malaria transmission enhanced by irrigated sites is imperative. It is important to maximize impact of available malaria control by ensuring adequate LLIN coverage and access to prompt treatment. Other additional measures may include larval source reduction through careful water resource management and larviciding of larval habitats, and indoor residual spray of insecticide for households near irrigation schemes among others. Future studies are needed to evaluate the impact of a community-led implementation of these control strategies on larval and adult vector abundance and malaria risk within irrigated agroecosystems.
